# Impact of diabetes type II and chronic inflammation on pancreatic cancer

**DOI:** 10.1186/s12885-015-1047-x

**Published:** 2015-02-13

**Authors:** Dietmar Zechner, Tobias Radecke, Jonas Amme, Florian Bürtin, Ann-Christin Albert, Lars Ivo Partecke, Brigitte Vollmar

**Affiliations:** 1 Institute for Experimental Surgery, Rostock University Medical Center, Schillingallee 69a, 18057 Rostock, Germany; 2 Department of General, Visceral, Thoracic and Vascular Surgery, University Medicine Greifswald, Ernst-Moritz-Arndt-University, Ferdinand-Sauerbruch-Straße, 17475 Greifswald, Germany

**Keywords:** Cancer stem cells, Cancer heterogeneity, Cancer cell plasticity, Aldh1, CD133

## Abstract

**Background:**

We explored if known risk factors for pancreatic cancer such as type II diabetes and chronic inflammation, influence the pathophysiology of an established primary tumor in the pancreas and if administration of metformin has an impact on tumor growth.

**Methods:**

Pancreatic carcinomas were assessed in a syngeneic orthotopic pancreas adenocarcinoma model after injection of 6606PDA cells in the pancreas head of either B6.V-Lep^ob/ob^ mice exhibiting a type II diabetes-like syndrome or normoglycemic mice. Chronic pancreatitis was then induced by repetitive administration of cerulein. Cell proliferation, cell death, inflammation and the expression of cancer stem cell markers within the carcinomas was evaluated by immunohistochemistry. In addition, the impact of the antidiabetic drug, metformin, on the pathophysiology of the tumor was assessed.

**Results:**

Diabetic mice developed pancreatic ductal adenocarcinomas with significantly increased tumor weight when compared to normoglycemic littermates. Diabetes caused increased proliferation of cancer cells, but did not inhibit cancer cell necrosis or apoptosis. Diabetes also reduced the number of Aldh1 expressing cancer cells and moderately decreased the number of tumor infiltrating chloracetate esterase positive granulocytes. The administration of metformin reduced tumor weight as well as cancer cell proliferation. Chronic pancreatitis significantly diminished the pancreas weight and increased lipase activity in the blood, but only moderately increased tumor weight.

**Conclusion:**

We conclude that diabetes type II has a fundamental influence on pancreatic ductal adenocarcinoma by stimulating cancer cell proliferation, while metformin inhibits cancer cell proliferation. Chronic inflammation had only a minor effect on the pathophysiology of an established adenocarcinoma.

## Background

Pancreatic cancer is one of the most lethal malignancies. The 5-year survival rate is despite therapeutic improvements still only 6% [1]. More than 80% of the pancreatic tumors are classified as pancreatic ductal adenocarcinoma (PDA). Novel therapies, but also the knowledge about pathophysiological factors influencing the progression of this malignant disease might help to find combinations of treatments to improve the survival rate. Key pathophysiological processes of cancer such as recurrence after chemotherapy and metastasis have been suggested to depend on cancer cell plasticity [2]. A prominent albeit controversial hypothesis, describing one form of cancer cell plasticity, is the concept of the existence of cancer stem cells (CSC) [2]. Cancer stem cells (CSC) are assumed to proliferate slowly, to have the capacity to renew themselves but also to give rise to distinct cell populations [3,4]. In PDA these cells have been reported to express specific genes such as Aldh1 or CD133 [5-9].

Much is known about factors increasing the likelihood to develop PDA. Identified risk factors include among others chronic pancreatitis, long lasting diabetes, and obesity [10]. Patients with chronic and especially hereditary pancreatitis have a very high relative risk of developing pancreatic cancer of 13.3 and 69.0, respectively [11]. Patients with diabetes and obesity have a moderately increased relative risk of 1.8 and 1.3 [12,13]. These studies indicate that a substantial number of patients with PDA also suffer from local inflammation or diabetes [10,14].

While some experimental studies exist that demonstrate that pancreatitis and diabetes influence potential precursor lesion of PDA such as PanINs or pancreatic duct glands [15-18], it is not known, if these factors also influence the pathophysiology of established carcinomas.

In order to evaluate if diabetes type II and inflammation influence the pathophysiology of PDA, we established a syngeneic orthotopic tumor model in mice and addressed the questions, if pancreatitis or diabetes type II influence cancer cell proliferation, cancer cell death, tumor-stroma interaction or the cancer stem cell compartment in these carcinomas.

## Methods

### Cell lines and cell culture

The cell lines, 6606PDA, 6606l and 7265PDA were a kind gift from Prof. Tuveson, Cambridge, UK. The 6606PDA and 6606l cell lines were originally isolated from a pancreatic adenocarcinoma or the respective liver metastasis of a mouse with C57BL/6J background, which expressed the KRAS^G12D^ oncogene in the pancreas (p48-cre induced expression of the oncogene) [19]. The 7265PDA cell line was isolated from a pancreatic adenocarcinoma of a mouse, which expressed the KRAS^G12D^ oncogene and in addition the p53^R172H^ allele in the pancreas (Pdx1-creER induced expression of the two alleles). All cell lines were maintained in DMEM high glucose medium with 10% fetal calf serum. For the injection of 6606PDA cells, subconfluent cultures of cells were trypsinized and the trypsinization was stopped by medium. After centrifugation the cells were resuspended in PBS, the suspension was mixed with an equal volume of Matrigel (BD Bioscience, San José, Calif., USA, Nr: 354248) and kept on ice (at a concentration of 1.25x10^7^ cells/ml) until injection [20]. For re-isolation of cells from carcinomas, tumors were isolated and cut up into small pieces. The pieces and outgrowing cells were cultivated in DMEM high glucose medium with 10% fetal calf serum.

### Evaluation of cells

Western blots were performed by separating cell lysate on SDS polyacryl gels and transferring the proteins to a polyvinyldifluoride membrane (Immobilon-P; Millipore, Eschborn, Germany). The membranes were blocked with 2.5% (wt/vol.) BSA or 5% (wt/vol.) milk powder (for the analysis of CD133) and incubated overnight at 4°C with a rabbit anti-ALDH1a1 (Cell Signaling, Boston, USA, code 12035, 1:1000), rat anti-CD133 (eBioscience Inc., San Diego, USA, code 14-1331, 1:500) or goat anti-GFAP (Abcam, Cambridge, UK, code ab53554,1:2000) antibody followed by incubation with a secondary peroxidase-linked anti-rabbit antibody (Cell Signaling, code 7074, 1:1000), anti-rat antibody (Santa Cruz Biotechnology, Santa Cruz, USA, code sc3823, dilution 1:10,000), or anti-goat (Santa Cruz Biotechnology, sc-2020, 1:20.000). For analysis of β-actin production, membranes were stripped, blocked by 2.5% (wt/vol.) BSA and incubated with mouse anti-β-actin antibody (Sigma-Aldrich, St Louis, MO, code A5441, dilution 1:20000) followed by peroxidase-linked anti-mouse antibody (Sigma-Aldrich, USA; code A9044, dilution 1:60,000). Protein production was visualized by luminol-enhanced chemiluminescence (ECL plus; GE Healthcare, Munich, Germany) and digitalised with Chemi- Doc XRS System (Bio-Rad Laboratories, Munich, Germany). Signals were densitometrically assessed and corrected with the signal intensity of β-actin (Quantity One; Bio-Rad Laboratories).

For the analysis of CD133 mRNA by PCR total RNA from cells or kidney was isolated using a RNeasy Mini Kit (Qiagen, Germany) according to the manufacturer‘s instructions. After a quality control of the isolated RNA by agarose gel electrophoresis first strand cDNA was synthesized by reverse transcription of 2 μg of total RNA using oligo(dT)18 primer (Biolabs, Frankfurt am Main, Germany) and Superscript II RNaseH-Reverse Transcriptase (Invitrogen, Karlsruhe, Germany). After heat inactivation of the reverse transcriptase 1/20 of the cDNA was amplified (27 cycles: 94°C for 30, 68°C for 40, 72°C for 60 seconds) using CD133 specific primers (forward primer: CCCTCCAGCAAACAAGCAAC, reverse primer: ACAGCCGGAAGTAAGAGCAC) and the PCR product of 325 bp was visualized by agarose gel electrophoresis.

For the quantification of cell proliferation rates, cells were plated on 96 well plates, so that the cells were 20% confluent, when BrdU was added to the medium. The BrdU incorporation was measured after 24 hours of incubation by the colorimetric cell proliferation assay as specified by the manufacturer (Roche Applied Science, Penzberg, Germany).

### Animals

For this study male B6.V-Lep^ob/ob^ mice (obese mice) were compared with male B6.V-Lep^+/?^ littermates (lean mice). The therapy with metformin was performed on male C57BL/6J mice. The mouse strains were originally purchased from The Jackson Laboratory (Bar Harbor, ME) and bred in our local animal facility. For defining the border between carcinoma and the desmoplastic reaction, carcinoma cells were injected in the pancreas of C57BL6-Tg^ACTB-eGFP1Osb/J^ mice (with a corresponding phenotype to lean B6.V-Lep^+/?^ mice) [21]. Animals were kept on water and standard laboratory chow ad libitum. All experiments were executed in accordance with the EU-directive 2010/63/EU and approved by the Landesamt für Landwirtschaft, Lebensmittelsicherheit und Fischerei Mecklenburg-Vorpommern (7221.3-1.1-069/12).

### Syngeneic orthotopic carcinoma model

For injection of carcinoma cells general anesthesia was induced in 93 ± 32 day old mice (average ± standard deviation) by 1.2-2.5% isoflurane. Perioperative analgesia was ensured by sc injection of 5mg/kg carprofen (Rimadyl, Pfizer GmbH, Berlin, Germany) and eyes were protected by eye ointment. After shaving and disinfection of the skin, the abdominal cavity was opened by transverse laparotomy and the head of the pancreas was identified. Duodenum and pancreas was gently lifted by tweezers and 20 μl cell suspension containing 2.5x10^5^ carcinoma cells were injected slowly into the head of the pancreas using a precooled ga22s 710 RN 100 ul syringe (Hamilton Syringe, Reno, Nev., USA). The pancreas was placed back into the abdominal cavity and the cavity was closed by a coated 5-0 vicryl suture (Johnson & Johnson MEDICAL GmbH, Norderstedt, Germany). The skin was then closed by a 5-0 prolene suture (Johnson & Johnson MEDICAL GmbH). On day 8 after the injection of carcinoma cells, chronic pancreatitis was induced over 2 weeks by administration of three ip injections of 50 μg/kg cerulein (Sigma-Aldrich Chemie GmbH), 3 days a week, at a rate of one every hour per day. Control mice were sham treated appropriately with 0.9% saline solution instead of cerulein and tissues were analyzed on day 20. For the evaluation of the impact of metformin on cancer pathophysiology 250 mg/kg 1,1-dimethylbiguanide hydrochloride (Sigma-Aldrich, code 150959) was ip injected daily from day 8 to day 15 followed by daily injection of half of this dose from day 16 to day 29 and analysis of the tumor on day 29 (3-6 hours after the last metformin administration). Control mice were sham treated appropriately with PBS instead of metformin and tumors were analyzed on day 29. For pain relief, 800 mg/L metamizol (Ratiopharm GmbH, Ulm, Germany) was added to the drinking water during the entire timespan of all in vivo experiments. In order to assess cell proliferation 50 mg/kg 5-bromo-2-deoxyuridine (BrdU) was injected ip 2.5 hours before tissue asservation. For blood samples and organ harvest, animals were anesthetized with 90 mg/kg ketamine (bela-pharm, Vechta, Germany) and 7 mg/kg xylazine (Bayer Health Care, Leverkusen, Germany).

### Analysis of the blood

Blood glucose concentrations were measured with the blood glucose meter Contour (Bayer Vital, Leverkusen, Germany) on day 0 before injection of carcinoma cells and on day 20 before the first cerulein injection of this day. Blood samples for assessing lipase activity were taken two hours after the third cerulein injection on day 8. The activity of lipase in blood plasma was analysed using the Cobas c111 spectrophotometer (Roche Diagnostics, Mannheim, Germany).

### Evaluation of tissue

The pancreas and tumor weight was measured after careful separation of the carcinoma from the pancreas. Evaluation of CD133 expression was performed on 7 μm cryo-sections. These sections were fixed with 4% paraformaldehyde in PBS for 15 min, reactive groups were then quenched in 50 mM NH4Cl for 10 min and the cell membranes were permeabilised with 0.3% saponin in PBS for 15 min, before CD133 immunohistochemistry was performed. All other data were obtained on 4μm paraffin sections after fixing the tissue in 4% (wt/vol.) phosphate-buffered formalin for 2–3 days. Histology was evaluated after staining paraffin sections with haematoxylin and eosin (H/E). Planimetric analysis of necrotic areas was performed on 10 randomly chosen pictures (taken with a 20x objective) of each carcinoma by using Adobe Photoshop CS5 (Adobe, San Jose, CA, USA). Apoptosis was analysed using the ApopTag Plus Peroxidase in situ detection kit (Millipore, Eschborn, Germany). To evaluate the cellular inflammatory response to cerulein injection, naphthol AS-D chloroacetate esterase (CAE) staining was performed on sections. Cell proliferation, chronic pancreatitis, and desmoplastic reaction were evaluated by immunohistochemistry using mouse anti-BrdU (Dako, Hamburg, Germany, clone Bu20a, dilution 1:50), rabbit anti-collagen-I (Abcam, code ab 34710, dilution 1:200), or rabbit anti-α-smooth muscle actin (Abcam, code ab5694, dilution 1:800) antibody. To verify desmoplastic reaction by the host, carcinoma cells were assessed in GFP expressing mice with goat anti-GFP antibody (Gene Tex, San Antonio, Texas, USA, GTX26673, 1:500). Cancer cells were further characterized by immunohistochemistry using rabbit anti-ALDH1a1 (Cell Signaling, code 12035, 1:800), goat anti-GFAP (Abcam, code ab7260,1:2000) or rat–anti CD133 (a generous gift by Denis Corbeil, Dresden, Germany, 1:200). Additional immunohistochemistry was performed using rat-anti-cytokeratin 19 (The Developmental Studies Hybridoma Bank at the University of Iowa, Iowa City, USA, clone TROMA-III, dilution 1:50), rat anti-F4/80 (AbD Serotec, Oxford, UK, MCA497, 1:10) or goat anti-vimentin (Santa Cruz Biotechnology, Santa Cruz, USA, sc7557, dilution 1:50) antibody. The following secondary antibodies were used: the Universal LSAB^+^ Kit/HRP (Dako) for primary goat, rabbit or mouse antibodies or alkaline phosphatase conjugated anti-rat (Santa Cruz Biotechnology, sc2021, 1:200) antibody for primary rat antibodies. All quantifications of cells or of necrotic areas were performed 120 to 270 μm from the tumor margin.

### Statistics

Data presentation and statistics were performed as described previously [15]. The significance of differences was evaluated using a Mann-Whitney rank-sum test, followed by the correction for the accumulation of the α error by considering the number of meaningful comparisons. Differences with P ≤ 0.05, divided by the number of meaningful comparisons, were considered to be significant.

## Results

### Characterisation of the syngeneic orthotopic carcinoma model

**Figure 1:**
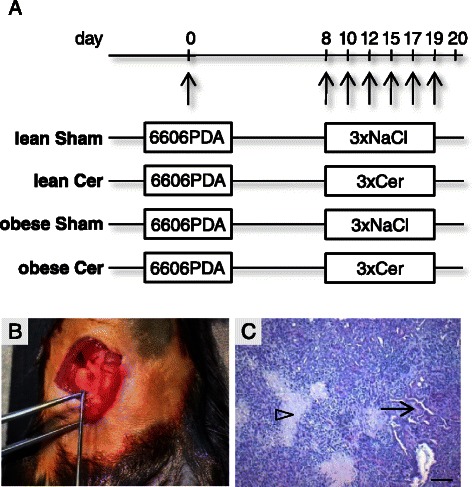
**Characterisation of the syngeneic orthotopic PDA model. (A)** 6606PDA cells were injected on day 0 into the head of the pancreas of non-diabetic (lean) or diabetic (obese) mice. Chronic pancreatitis was induced by ip injection of cerulein in non-diabetic (lean Cer) and diabetic (obese Cer) cohorts of mice three times a day on the indicated days, whereas control non-diabetic (lean Sham) and diabetic (obese Sham) mice received 0.9% saline solution. Tissue samples were analyzed on day 20. **(B)** The correct injection of carcinoma cells could macroscopically be verified. **(C)** A representative histology of a PDA reveals necrotic areas (arrowhead), but also vital cells with partially epithelial morphology (arrow). Bar = 50 μm.

**Figure 2:**
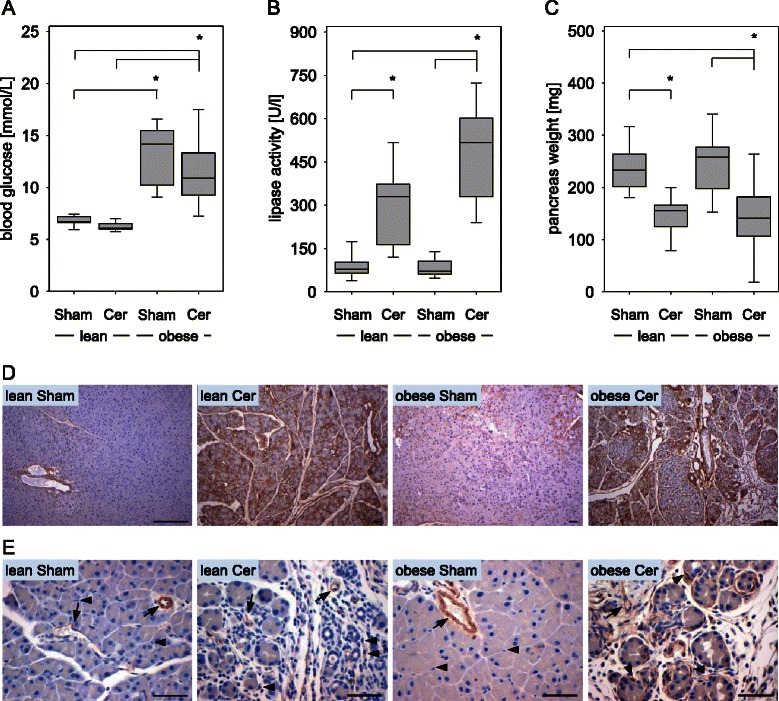
**Characterisation of diabetes and pancreatitis. (A)** The average blood glucose concentration of two measurements per mouse (day 0 and day 20) for each cohort is given for sham treated non-diabetic (lean Sham) or diabetic (obese Sham) mice and in cerulein treated non-diabetic (lean Cer) or diabetic (obese Cer) animals. **(B)** Comparison of the lipase activity in the blood between the four cohorts indicates induction of pancreatitis on day 8. **(C)** Evaluation of the pancreas weight on day 20 indicates pancreatic atrophy after induction of chronic pancreatitis. **(D)** Immunohistochemistry on day 20 indicates collagen I deposition (brown colour) in the pancreas after cerulein induced chronic pancreatitis in lean and obese mice. **(E)** Evaluation of α-smooth muscle actin expression by immunohistochemistry (brown colour) on day 20 indicates moderate activation of periacinar stellate cells by cerulein in lean mice and strong activation in obese mice (arrows point at blood vessels, arrowheads point at stellate cells). Box plots indicate the median, the 25^th^ and 75^th^ percentiles in the form of a box, and the 10^th^ and 90^th^ percentiles as whiskers. The number of animals evaluated was n = 11 (lean Sham), n = 10 (lean Cer), n = 11 (obese Sham), n = 13 (obese Cer). Significant differences between the cohorts are indicated, *P ≤ 0.006. Bars = 50 μm.

To test whether diabetes, chronic pancreatitis or a combination of both influence the pathophysiology of a fully established PDA, we injected 6606PDA cells into the head of the pancreas in either diabetic mice (obese) or normoglycemic (lean) littermates (Figure 1A and B). Administration of cerulein (Cer) or saline (Sham) in both genotypes allowed us to compare pathophysiological parameters in carcinoma during pancreatitis (lean, Cer), diabetes (obese, Sham), or diabetes with concurrent pancreatitis (obese, Cer) to carcinoma in animals without diabetes or pancreatitis (lean, Sham). We observed that independent of treatment or genotype 100% of mice developed a carcinoma within 20 days. Histological analysis of the carcinomas revealed vital tissue with partial epithelial morphology, but also necrotic areas within the tumor (Figure 1C). Obese mice had significantly increased blood glucose concentrations, when compared to lean littermates (Figure 2A). Successful induction of pancreatitis by cerulein administration was verified by increased lipase activity and reduced pancreas weight in cerulein treated obese as well as lean mice when compared to sham treated controls (Figure 2B and C). In addition, cerulein administration causes the deposition of collagen I (Figure 2D) and the expression of α-smooth muscle actin in periacinar stellate cells (Figure 2E) of lean as well as obese mice when compared to sham treated animals. The induction of α-smooth muscle actin in cerulein treated lean mice, however, was weaker when compared to cerulein treated obese mice (Figure 2E).

### Diabetes increases tumor size and proliferation of carcinoma cells

**Figure 3:**
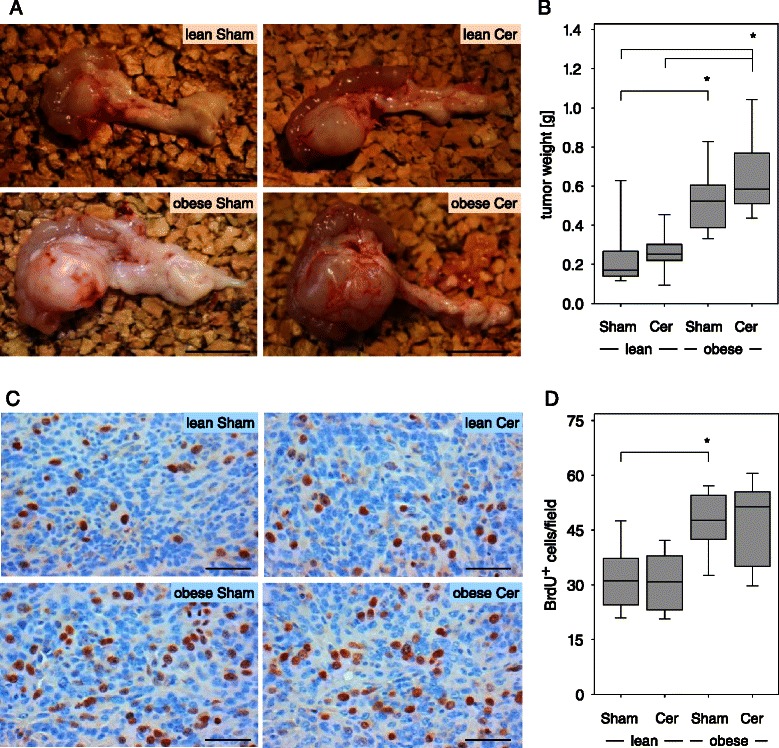
**Diabetes leads to increased tumor weight and enhanced cancer cell proliferation on day 20. (A)** Representative images of isolated pancreas with a carcinoma shows obvious differences in tumor size in sham treated non-diabetic (lean Sham) or diabetic (obese Sham) mice and in cerulein treated non-diabetic (lean Cer) or diabetic (obese Cer) animals. **(B)** Quantification of the tumor weight in the indicated mouse cohorts. **(C)** Representative images of histological sections after BrdU immunohistochemistry. **(D)** Quantification of BrdU^+^ nuclei within the carcinoma reveals increased proliferation of cancer cells in diabetic mice. Box plots indicate the median, the 25^th^ and 75^th^ percentiles in the form of a box, and the 10^th^ and 90^th^ percentiles as whiskers. The number of animals evaluated was n = 11 (lean Sham), n = 10 (lean Cer), n = 11 (obese Sham), n = 13 (obese Cer). Significant differences between the cohorts are indicated, *P ≤ 0.002 (B), *P = 0.005 (D). Bar =1 cm (A) or 50 μm (C).

Within three weeks after the injection of adenocarcinoma cells in the pancreas diabetic obese mice developed tumors, which were obviously larger than the tumors in normoglycemic lean littermates (Figure 3A). Measuring the tumor weight revealed significantly larger carcinomas in sham treated obese mice, when compared to sham treated lean littermates (Figure 3B). Increased tumor weight was also observed in cerulein treated obese mice when compared to cerulein or sham treated lean littermates (Figure 3B). Only a moderate increase in tumor weight was observed in cerulein treated obese or lean mice when compared to the same genotype of mice, which received sham treatment (Figure 3B).

To evaluate if diabetes modulates proliferation of cancer cells, the number of BrdU^+^ cells within the carcinoma were evaluated (Figure 3C). Proliferation of cancer cells was significantly increased in sham treated obese mice when compared to sham treated lean littermates (Figure 3D). Increased proliferation was also observed in cerulein treated obese mice when compared to cerulein or sham treated lean littermates (Figure 3D). These data suggest that in mice with a diabetes type II like syndrome carcinoma cells have a higher proliferation rate resulting in increased tumor size. In order to evaluate, if the intrinsic growth ability of cancer cells changes permanently in obese mice, we re-isolated the cancer cells from carcinomas in lean and obese mice and compared their proliferation rate in vitro. Carcinoma cells, which were isolated from lean mice, had a very similar proliferation rate to carcinoma cells, which were isolated from obese mice (lean: 1.09/1.06-1.14, n = 3; obese: 1.06/0.96-1.21, n = 6), or 6606PDA cells, which were never injected in any animal (1.05/0.99-1.21, n = 7; median/interquartile range of BrdU incorporation measured by ELISA). Thus, diabetes does not (e.g. via epigenetic mechanisms) permanently change the proliferative capacity of tumor cells.

### Diabetes does not decrease cell death in carcinomas

**Figure 4:**
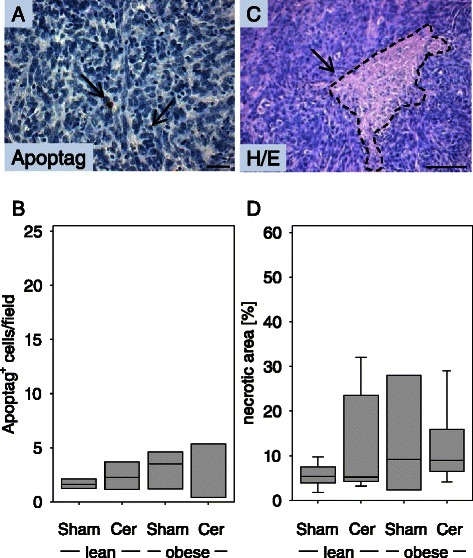
**Diabetes does not inhibit cell death in PDA on day 20. (A)** Representative image of an Apoptag^+^ cell. **(B)** Quantification of apoptotic cell death in the carcinomas of sham treated non-diabetic (lean Sham) or diabetic (obese Sham) mice and in the carcinomas of cerulein treated non-diabetic (lean Cer) or diabetic (obese Cer) animals. **(C)** Representative image of a necrotic area. **(D)** Comparison of the percentage of necrotic tissue area in the carcinomas of the indicated mouse cohorts. Box plots indicate the median, the 25^th^ and 75^th^ percentiles in the form of a box, and the 10^th^ and 90^th^ percentiles as whiskers. The number of animals evaluated was n = 4 (lean Sham), n = 4 (lean Cer), n = 3 (obese Sham), n = 4 (obese Cer) in panel B and n = 7 (lean Sham), n = 7 (lean Cer), n = 3 (obese Sham), n = 6 (obese Cer) in panel D. Differences between the cohorts were not significant. Bar = 50 μm.

In order to evaluate apoptosis, Apoptag^+^ cells were quantified within carcinomas. No obvious decrease in the number of Apoptag^+^ cells in diabetic mice could be observed when compared to nondiabetic littermates (Figure 4A and B). Planimetric analysis of H/E stained histological sections revealed that diabetes did also not reduce the relative area of necrosis within the carcinomas (Figure 4C and D).

### Characterisation of the cancer stem cell compartment

**Figure 5:**
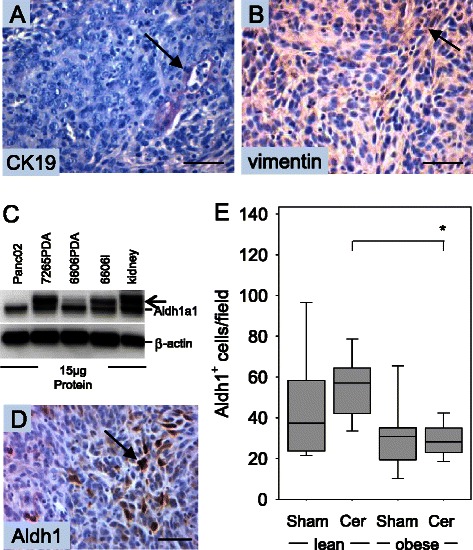
**Analysis of CK19, vimentin and Aldh1a1 expression. (A)** Representative images of epithelial cells expressing cytokeratin 19 and **(B)** of non-epithelial cells expressing vimentin in 6606PDA derived carcinomas. **(C)** Analysis of Aldh1a1 expression in cultured PDA cell lines and kidney by Western Blotting. An additional band (arrow) is observed in some cell lines and kidney cell extract and might represent another Aldh family member; e.g. Aldh1a3. **(D)** Immunohistochemistry of 6606PDA derived carcinomas reveals expression of the cancer stem cell marker, Aldh1, in some cancer cells. **(E)** Quantification of Aldh1^+^ cells in the carcinomas of sham treated non-diabetic (lean Sham) or diabetic (obese Sham) mice and in the carcinomas of cerulein treated non-diabetic (lean Cer) or diabetic (obese Cer) animals. Box plots indicate the median, the 25^th^ and 75^th^ percentiles in the form of a box, and the 10^th^ and 90^th^ percentiles as whiskers. The number of animals evaluated was n = 9 (lean Sham), n = 9 (lean Cer), n = 9 (obese Sham), n = 10 (obese Cer). Significant differences between the cohorts are indicated, *P = 0.003. The Western Blot results were reproduced by three independent experiments. Bars = 50 μm.

Cytokeratin 19 and vimentin expression was analysed in the carcinomas in order to evaluate if injected cancer cells can give rise to distinct cell types. In tumors, cells with epithelial morphology expressed the epithelial marker cytokeratin 19 (Figure 5A), whereas non-epithelial cells expressed the mesenchymal marker vimentin (Figure 5B). These data suggest that injected cancer cells can differentiate into at least two different cell types, and that a pluripotent cell population might be present within the injected cancer cells. To evaluate if pancreatic cancer cell lines express cancer stem cell markers such as Aldh1 we compared the expression of Aldh1a1 in pancreatic cancer cell lines such as Panc02, 7265PDA and 6606PDA with the liver metastasis cell line 6606l. The Aldh1a1 protein was readily observed with an apparent molecular weight of 55 kDa in all cell lines as well as in kidney cell extract, used as a positive control (Figure 5C). In some cell lines the antibody also detected another protein with an apparent molecular weight of 58 kDA, which is possibly Aldh1a3 or another Aldh family member (Figure 5C). In carcinomas few cells specifically expressed Aldh1 as evaluated by immunohistochemistry (Figure 5D). The number of Aldh1^+^ cells was moderately decreased in sham treated obese mice when compared to sham treated lean littermates (Figure 5E). A significantly decreased number of Aldh1^+^ cells was also observed in cerulein treated obese mice when compared to cerulein treated lean littermates (Figure 5E).

**Figure 6:**
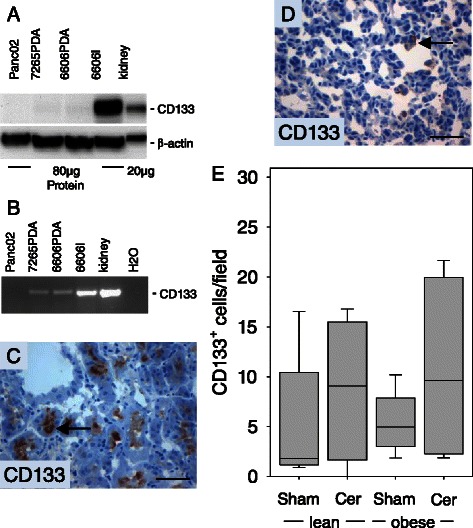
**Analysis of CD133 expression. (A)** Analysis of CD133 expression in cultured 6606PDA cells and kidney by Western Blotting. **(B)** Analysis of CD133 expression in cultured PDA cell lines and kidney by PCR. **(C)** The positive control for CD133 immunohistochemistry reveals expression of CD133 (arrow) in epithelial cells of proximal tubuli. **(D)** Immunohistochemistry of 6606PDA derived carcinomas reveals expression of CD133 (arrow) in some cancer cells. **(E)** Quantification of CD133^+^ cells in the carcinomas of sham treated non-diabetic (lean Sham) or diabetic (obese Sham) mice and in the carcinomas of cerulein treated non-diabetic (lean Cer) or diabetic (obese Cer) animals. Box plots indicate the median, the 25^th^ and 75^th^ percentiles in the form of a box, and the 10^th^ and 90^th^ percentiles as whiskers. The number of animals evaluated was n = 5 (lean Sham), n = 4 (lean Cer), n = 6 (obese Sham), n = 6 (obese Cer). Differences between the cohorts were not significant. The Western Blot results were reproduced by three independent experiments. Bars = 50 μm.

We also characterized the expression of an additional cancer stem cell marker, CD133. This protein was not detected in the Panc02, 7265PDA and 6606PDA cell lines by Western Blotting, but was highly expressed in the 6606l cell line and kidney (Figure 6A). However, since a low level of CD133 mRNA could be detected in 7265PDA and 6606PDA cells by PCR (Figure 6B), we evaluated if a few CD133^+^ cells could be observed in 6606PDA cell derived carcinomas. CD133 expression could be easily observed on the apical membrane of epithelial cells lining the proximal tubuli of the kidney as published previously (Figure 6C) [22]. CD133^+^ cells could also be observed in few cells of 6606PDA derived carcinomas (Figure 6D). The number of CD133^+^ cells was moderately increased in cerulein treated lean mice when compared to sham treated lean littermates (Figure 6E). A moderately increased number of CD133^+^ cells was also observed in cerulein treated obese mice when compared to cerulein treated lean littermates (Figure 6E). We also analyzed the expression of GFAP, a protein expressed by glioblastoma and neural stem cells. GFAP was easily detected by Western Blotting in Panc02, 7265PDA, 6606PDA, 6606l cells and brain, but only elusive expression was observed in 6606PDA cell derived carcinomas by immunohistochemistry (data not shown).

### Evaluation of inflammation and the desmoplastic reaction

**Figure 7:**
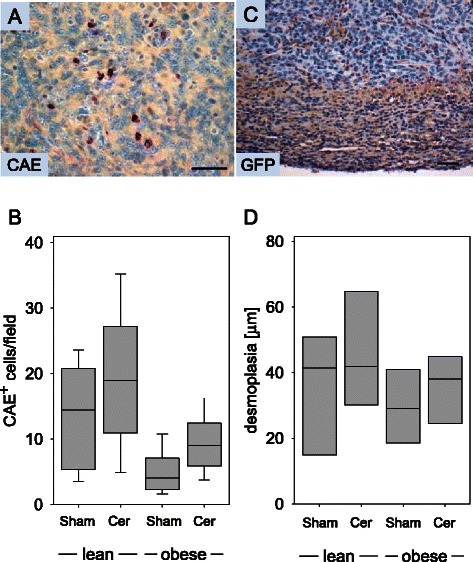
**Analysis of inflammation and desmoplasia on day 20. (A)** Representative image of CAE^+^ inflammatory cells in PDA. **(B)** Quantification of CAE^+^ cells in the carcinomas of sham treated non-diabetic (lean Sham) or diabetic (obese Sham) mice and in the carcinomas of cerulein treated non-diabetic (lean Cer) or diabetic (obese Cer) animals. **(C)** Desmoplastic reaction visualized by anti-GFP immunohistochemistry in a C57BL6-Tg^ACTB-eGFP1Osb/J^ mouse, which ubiquitously expresses GFP. **(D)** Quantification of α-smooth muscle^+^ desmoplastic reaction surrounding the carcinomas in sham treated non-diabetic (lean Sham) or diabetic (obese Sham) mice and in the carcinomas of cerulein treated non-diabetic (lean Cer) or diabetic (obese Cer) animals. Box plots indicate the median, the 25^th^ and 75^th^ percentiles in the form of a box, and the 10^th^ and 90^th^ percentiles as whiskers. The number of animals evaluated was n = 11 (lean Sham), n = 9 (lean Cer), n = 9 (obese Sham), n = 12 (obese Cer) in Panel B and n = 4 for each cohort in Panel D. Bar = 50 μm.

Since surprisingly little influence of pancreatitis on the pathophysiology of PDA was observed in our study, we evaluated, if pancreatitis lead to more infiltrating inflammatory cells in the carcinoma. Because cerulein induced pancreatitis is characterized mainly by infiltrating neutrophil granulocytes, the number of CAE^+^ cells was evaluated (Figure 7A and B). Indeed, a moderately increased number of CAE^+^ cells was detected in the carcinomas of cerulein treated mice compared to sham treated animals (Figure 7B). Diabetes, however, caused a small reduction in the number of tumor infiltrating CAE^+^ granulocytes. The observed differences were not significant. Similarily, a moderately increased number of F4/80^+^ mahrophages was detected in the carcinomas of cerulein treated mice compared to sham treated animals (data not shown). This suggests that strong inflammation in the pancreas did not automatically lead to a major increase in the number of inflammatory cells in the tumor. To verify, if a desmoplastic reaction by the host might shield the carcinomas, we injected the 6606PDA cells in C57BL6-Tg^ACTB-eGFP1Osb/J^ mice expressing GFP ubiquitously. We observed that carcinomas were surrounded by GFP^+^ fibroblast like cells (Figure 7C). Quantification of the thickness of the α-smooth muscle actin positive desmoplastic reaction surrounding the carcinomas, revealed a moderate increase in the thickness of the desmoplastic reaction in cerulein treated mice when compared to sham treated animals (Figure 7D). In diabetic mice this desmoplastic reaction was moderately reduced (Figure 7D).

### Metformin decreases tumor size and proliferation of carcinoma cells

**Figure 8:**
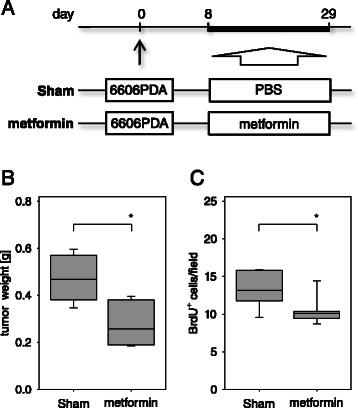
**Metformin reduces tumor weight and cancer cell proliferation. (A)** 6606PDA cells were injected on day 0 into the pancreas of C57BL/6J mice. Between day 8 and day 29 PBS (Sham) or metformin was ip injected daily and tissue samples were analyzed on day 29. **(B)** Quantification of the tumor weight in the indicated mouse cohorts. **(C)** Quantification of BrdU^+^ nuclei within the carcinoma reveals that metformin treatment reduces cell proliferation. Box plots indicate the median, the 25^th^ and 75^th^ percentiles in the form of a box, and the 10^th^ and 90^th^ percentiles as whiskers. The number of animals evaluated was n = 8 (Sham) and n = 7 (metformin), Significant differences between the cohorts are indicated, *P = 0.004 **(B)**, *P = 0.029 **(C)**.

In order to evaluate if the antidiabetic drug, metformin, has an effect on PDA, we injected 6606PDA cells into the head of the pancreas on day 0. From day 8 to 29 one mouse cohort was sham treated, whereas the other cohort was treated with metformin (Figure 8A). Measuring the tumor weight on day 29 revealed significantly smaller carcinomas in metformin treated mice, when compared to sham treated littermates (Figure 8B). The proliferation of cancer cells was also significantly decreased in metformin treated mice when compared to sham treated littermates (Figure 8C). These data suggest that metformin reduces the proliferation rate of carcinoma cells resulting in smaller tumors.

## Discussion

The presented data demonstrate that a diabetes type II like syndrome i) increases the weight of PDA, ii) stimulates the proliferation of cancer cells, iii) does not inhibit the cell death of cancer cells and iv) reduces the number of Aldh1^+^ cells within the tumor. We observed, however, no major influence of chronic pancreatitis on the pathophysiology of PDA. In addition presented data demonstrate that the antidiabetic drug metformin i) decreases the weight of PDA and ii) reduces the proliferation of cancer cells.

The observed major effect in B6.V-Lep^ob/ob^ mice on the pathophysiology of PDA might be caused by distinct features of these mice such as hyperinsulinaemia, hyperglycaemia or by adipositas. These features are typical for the early stage of type II diabetes. Alternatively, hyperglycaemia and adipositas are also associated with the metabolic syndrome. Indeed, this mouse strain has been used as model system for both diseases [23,24]. Nevertheless, we favor the idea, that this mouse strain is a model for type II diabetes rather than for the metabolic disease, since B6.V-Lep^ob/ob^ mice do have increased high-density lipoprotein concentrations and are thus protected from diet-induced atherosclerosis [23]. Since this mouse strain is characterized by a 10-fold higher C-peptide level but by only about a 2-fold higher glucose concentration in the blood [15], it is likely that hyperinsulinaemia rather than hyperglycemia stimulates cancer cell proliferation and increases the tumor weight. This hypothesis is supported by experimental studies indicating that streptozotocin induced type I like diabetes, which is characterized by higher blood glucose concentration but lower insulin concentration, does not increase tumor weight in pancreatic carcinomas [25]. In addition, the observation that human pancreatic cancer cell lines express insulin receptor and that the proliferation of these cell lines is induced by insulin also supports this conclusion [26,27]. High concentrations of insulin can also activate the IGF-1 receptor [28]. This receptor and its adaptor proteins IRS-1 and IRS-2 are expressed in pancreatic cancer cell lines as well as in human PDA [29-31]. These data underscore the importance of these signaling pathways in promoting pancreatic cancer cell proliferation and suggest that blocking the IGF-I receptor might be a valuable approach for targeted therapy of pancreatic cancer. In fact, Ganitumab, a monoclonal IGF-1 receptor antibody, inhibited growth of pancreatic carcinoma xenografts in mice and showed tolerable toxicity and trends toward an improved 6-month survival rate in patients with metastatic pancreatic cancer [32,33].

Our data indicate that diabetes type II like syndrome increases tumor weight, but at the same time decreases the number of Aldh1^+^ cells. Since Aldh1 expression is a feature of cancer stem cells in pancreatic carcinoma [7-9,34,35], it is tempting to speculate that diabetes induces the differentiation of Aldh1^+^ quiescent cancer stem cells into fast proliferating Aldh1^−^ cells, which might contribute to increased tumor weight. This interpretation is consistent with the concept of quiescence of cancer stem cells and some adult stem cells [36,37]. Interestingly, insulin/IGF receptor signaling has been reported to abrogate the quiescent state of stem cells, which underlines our hypothesis that hyperinsulinemia increases cell proliferation in PDA [38]. However, it is also possible that Aldh1^+^ cells might not be true cancer stem cells in this animal model and that the reduced number of Aldh1^+^ cells in diabetic mice reflects how diabetes influences cancer cell plasticity in a cancer stem cell independent manner. It is also worth noticing that another so called tumor stem cell marker, CD133, can be observed in the carcinomas, but that the quantification of CD133^+^ cells does also not correlate well with tumor size. Since it has been suggested that chemotherapy resistance might be caused by cancer stem cells, we compared the expression of CD133 and Aldh1 between three gemcitabine resistant 6606PDA clones and the original gemcitabine sensitive 6606PDA cell line. These gemcitabine resistant clones did not express higher levels of Aldh1 or CD133 (data not shown). Possibly, Aldh1 or CD133 expression does not in all cases directly correlate with tumor size or chemoresistance and might also not always define cells with stem cell properties [39]. This interpretation is supported by the fact that these proteins are readily expressed by fully differentiated cells. For example, CD133 is expressed in epithelial cells of proximal tubuli in the kidney and Aldh1 is highly expressed in the epithelium of the intestine, in liver and in pancreas [22,40].

In contrast to diabetes type II, chronic pancreatitis had little influence on the pathophysiology of the carcinomas. This result is surprising considering the published consensus that chronic inflammation is a major risk factor for the development of PDA [10,11,14]. The following interpretations may explain this discrepancy: i.) Chronic inflammation might promote cancerogenesis at an early stage of PDA development, but might have little influence on advanced adenocarcinomas. This interpretation is consistent with data indicating that chronic inflammation increases the risk for developing precancerous lesions and PDA in humans as well as in genetically modified mice [10,16,17]. This hypothesis is also supported by publications, which demonstrate that anti-inflammatory drugs delay the progression of pancreatic cancer precursor lesions [41], but fail to have any benefit in the therapy of PDA [42]. ii.) Alternatively, chronic pancreatitis had little influence on the pathophysiology of the carcinomas, because of limitations of our animal model. Although we were able to induce a strong chronic pancreatitis by redundant administration of cerulein (Figure 2 B-E), we observed some local inflammation adjacent to the carcinoma and a strong desmoplastic reaction, independent of the induction of pancreatitis (data not shown and Figure 7). Possibly, this desmoplastic reaction shields the carcinomas from local inflammation or the observed peritumoral pancreatitis may have blunted the effects of cerulein. If the pathophysiology of a fully established pancreatic adenocarcinoma is influenced by pancreatitis or intra- and peritumoral inflammation, which is detected in most PDAs, is currently of intellectual as well as of clinical interest [14]. Our data indicate that a strong inflammatory milieu does not automatically lead to major changes in cancer cell proliferation, cell death or tumor size. However, a similar study with genetically modified mouse models of PDA needs to be pursued in order to exclude the possibility, that the observed effects are mouse model specific.

## Conclusion

In conclusion, these experiments provide support for the concept that a diabetes type II like syndrome promotes growth of PDA, whereas strong inflammation does not have a major influence on the pathophysiology of advanced PDA. Our data also demonstrate that an anti-diabetic medication such as metformin has anti-tumorigenic properties, which is consistent with recently published data on human PDA cells [43,44]. If this anti-tumorigenic effect will also be observed in clinical trials, which are currently pursued, remains to be seen (https://clinicaltrials.gov/) [45]. In addition, modulation of inflammation for the therapy in pancreatic cancer is a goal in several clinical and preclinical studies [14]. However, our data support the idea that modulation of cell metabolism might be more promising than modulation of inflammation for the treatment of PDA [14,46].
